# Impact of co-infections and immune responses on clinical severity of human adenovirus 3 and 7 infections in hospitalized children with lower respiratory tract infections: a comparative study

**DOI:** 10.3389/fcimb.2024.1482787

**Published:** 2025-01-09

**Authors:** Xiaolin Ma, Yuting Wu, Ri De, Hailan Yao, Feng He, Yi Wang, Wei Wang, Chao Yan, Qinwei Song, Chunjie Guo, Li Wen, Linqing Zhao, Ling Cao, Chunmei Zhu

**Affiliations:** ^1^ Department of Respiratory Medicine, Children’ s Hospital Affiliated to Capital Institute of Pediatrics, Beijing, China; ^2^ Laboratory of Virology, Beijing Key Laboratory of Etiology of Viral Disease in Children, Capital Institute of Pediatrics, Beijing, China; ^3^ Laboratory of Biochemistry and Immunology, Capital Institute of Pediatrics, Beijing, China; ^4^ Department of Central Laboratory, Capital Institute of Pediatrics, Beijing, China; ^5^ Laboratory of Bacteria, Capital Institute of Pediatrics, Beijing, China; ^6^ Department of Clinical Laboratory, Children’ s Hospital Affiliated to Capital Institute of Pediatrics, Beijing, China

**Keywords:** human adenovirus serotypes 3, human adenovirus serotypes 7, lower respiratory tract infections, co-infections, immune responses, children

## Abstract

**Background:**

The pathogenic distribution of co-infections and immunological status of patients infected with human adenovirus serotypes 3 or 7 (HAdV-3 or HAdV-7) were poorly understood.

**Methods:**

This study involved a retrospective analysis of respiratory specimens collected from enrolled children with lower respiratory tract infections (LRTIs), positive for HAdV-3 or HAdV-7 from January 2017 to December 2019. Demographic data, clinical features, laboratory and radiographic findings were compared to delineate the impact of co-infections, and immune responses on clinical severity of HAdV-3 or HAdV-7 infections.

**Results:**

Among 1311cases enrolled, there were 66 infected with HAdV-3 and 58 with HAdV-7. HAdV-7-infected patients exhibited more prolonged fever (100% vs 89.4%, *p*=0.014), pneumonia (100% vs 89.4%, *p*=0.014), hypoxia (34.5% vs 12.1%, *p*=0.003), higher propensity for aspartate aminotransferase exceeding 80U/L (21.1% vs 4.7%, *p*=0.006), D-Dimer exceeding 1.65mg/L (64.9% vs 12.5%, *p*<0.001), consolidation (50.0% vs 27.4%, *p*=0.011), and pleural effusion (32.8% vs 6.5%, p<0.001), co-infections with *Mycoplasma pneumoniae* (77.1% vs 32.6%, *p*<0.001), and multiple infections (56.8% vs 41.3%, *p*=0.007), compared to those with HAdV-3 infections. Immune cell analysis indicated that HAdV-7 infections led to a more pronounced decrease in CD3+ T cells (1596.8 vs 2444.8 cells/𝛍l, p=0.042), CD8+ cytotoxic T cells (668.6 vs 774.0 cells/µl, *p*=0.045), and increased NK cell percentages (11.5% vs 9.0%, *p*=0.044) compared to HAdV-3 infections.

**Conclusions:**

Hospitalized children with HAdV-7-associated LRTIs exhibit greater severity, multiple infections, and significant potential for greater cellular immune dysregulation compared to those with HAdV-3 infection, indicating a more severe clinical course and distinct pathogenic profiles.

## Introduction

Human adenoviruses (HAdV), specifically serotypes 3 and 7 (HAdV-3 and HAdV-7), are recognized as significant pathogens in the etiology of lower respiratory tract infections (LRTIs) among children (Lu et al., 2013; Chen et al., 2022). These infections pose substantial health concerns, particularly when they manifest in severe forms such as pneumonia (Jain et al., 2015; Xie et al., 2019). These infections not only lead to high rates of hospital admissions but also present a considerable challenge in terms of management and prevention, due to their rapid transmission, potential to cause large-scale outbreaks, and the absence of effective antiviral drugs or vaccines. The situation is further complicated by the variability in serotypes, clinical presentations and the potential for severe complications, including respiratory failure and death. This severity is notably pronounced in densely populated regions such as China, where frequent outbreaks have been highlighted critical areas for healthcare focus and intervention (Xie et al., 2019; Li et al., 2021; Zou et al., 2021; Wang et al., 2022; Liu et al., 2023a).

While the direct impact of these adenovirus serotypes on the severity of disease has been documented more and more (Lin et al., 2017; Liu et al., 2022; Wang et al., 2023), a critical gap remains in understanding how co-infections and variations in immune response influence the severity of these infections. This gap underscores the need for in-depth research focused on the interplay between co-infecting pathogens and the host’s immune system, which can significantly affect disease outcomes. Infections with HAdV not only result in primary disease manifestations but are also frequently complicated by secondary bacterial or viral infections (Mao et al., 2022; Liu et al., 2023b). These co-infections can exacerbate the severity of the respiratory illness, resulting in more severe symptoms, an increased risk of complications, and prolonged hospital stays (Gao et al., 2020; Chen et al., 2024). Moreover, the immune response to these co-infections, which can vary significantly among individuals, especially in pediatric patients, plays a crucial role in the progression and outcome of the disease. A robust immune response might control the spread of the virus and secondary infections more effectively, while an inadequate or overly aggressive response may lead to worse clinical outcomes (Nazir and Metcalf, 2005).

Currently, the pathogenic distribution of co-infections and the immunological status of patients infected with HAdV-3 or HAdV-7 remains poorly understood. This study aims to elucidate the role of co-infections and immune responses in shaping the clinical severity of HAdV-3 and HAdV-7 infections among hospitalized children with LRTIs. Through this research, we bridge a gap in the literature and lay the groundwork for a comprehensive investigation into the complex dynamics of adenovirus infections compounded by co-infecting organisms and the corresponding immunological responses.

## Materials and methods

### Study design and patients

This retrospective study was conducted at Capital Institute of Pediatrics between January 2017 and December 2019. The cohort included children under 18 years diagnosed with LRTIs and confirmed to have HAdV-3 or HAdV-7 infections.

### Specimens collection

Respiratory specimens, including throat swabs, nasopharyngeal swabs, nasopharyngeal aspirates, and bronchoalveolar lavage fluid (BALF), were collected from children admitted with LRTIs within two days of admission. Specimens were immediately transported to the laboratory. Two microliters of Hank’s solution were added for suspension prior to centrifugation at 1,200 g for ten minutes. Screening for common respiratory pathogens, including viruses, bacteria, and fungi, was conducted by the laboratory.

### Detection of respiratory pathogens

For multiple pathogen nucleic acid screening, nucleic acids were extracted using the QIAamp^®^ Viral RNA Mini Kit (QIAGEN, Germany) from a 140 µl sample volume, with the total elution volume of 60µl stored at -80℃ from the supernatants, and used for screening encompassed influenza A and B, respiratory syncytial virus, HAdV, metapneumovirus, rhinovirus, bocavirus, parainfluenza virus, coronavirus, *Mycoplasma pneumoniae* (MP) and *chlamydia pneumoniae* by the NxTAGTM RPP assay (Luminex Molecular Diagnostics Inc., Toronto, Canada) according to the manufacturer’s protocol.

Respiratory bacteria and atypical pathogens including *Streptococcus pneumoniae*, *Hemophilus influenzae*, *Staphylococcus aureus*, *Methicillin resistant staphylococcus*, *Escherichia coli*, *Klebsiella pneumoniae*, *Pseudomonas aeruginosa*, *Acinetobacter baumannii*, *Stenotrophomonas maltophilia*, *Legionella pneumoniae*, *Chlamydia pneumoniae*, *Mycobacterium tuberculosis* complex and MP, were identified in specimens through the Pathogenic Bacteria Nucleic Acid Detection Kit adhering to the manufacturers’ protocols (CapitalBio Technology, Beijing, China). Cultures for bacteria and fungi were performed on sputum and BALF specimens.

### Genotyping of HAdV

HAdV-positive specimens underwent molecular genotyping for HAdV-3 and HAdV-7 using Polymerase chain reaction and sequencing of the penton base, hexon, and fiber genes, as described previously (Yang et al., 2021; Wu et al., 2022). Sequences were analyzed using the Sanger method on an ABI3730xl DNA analyzer (SinoGenoMax Co., Ltd, Beijing, China).

### Phylogenetic analysis

Chromas Lite 2.22 and NCBI BLAST facilitated initial sequence analysis. Editseq and Seqman (DNAStar) were utilized for sequence editing and alignment. Phylogenetic trees were constructed using MEGA X with Clustal W (Molecular Evolutionary Genetics Analysis Version X), employing the neighbor-joining method and bootstrap analysis with 1,000 replicates for node reliability assessment (Tamura et al., 2004; Kumar et al., 2018).

### Detection of lymphocyte subset, immunoglobulins and complement levels

Lymphocyte percentages and counts were determined using flow cytometry. Peripheral blood specimens (50 μl) were collected into ethylenediaminetetraacetic acid anticoagulation tubes and transferred to BD Trucount™ Tubes using reverse sampling for absolute counting. This was followed by the addition of 450 μl hemolysin, which was incubated in the dark for 15 minutes. Cell subset counts were obtained using a BD FACSCanto™ II and analyzed with BD FACSCanto™ software version 3.1(BD, USA). All procedures adhered to applicable guidelines and regulations.

Using the fully-automated biochemical analyzer (Siemens, Germany, BN II machine), children’s serum samples were serially diluted with a sample dilution solution, and subsequently, anti-human immunoglobulins and complement antibodies were added. Turbidity measurements were taken at a wavelength of 340 nm using the immunoturbidimetric method, and ultimately, quantitative assessments of immunoglobulins (IgG, IgA, IgM, IgE) and complement levels (C3, C4) were conducted.

### Data collection

Demographic data, clinical features, laboratory findings, treatment outcomes, and radiographic features were retrospectively derived from electronic medical chart after the patients’ discharge from the hospital. LRTIs encompassed bronchitis, bronchiolitis, and pneumonia. Pneumonia was characterized by the presence of consolidation (a dense or fluffy opacity with or without air bronchograms), other infiltrates (linear and pathy alveolar or interstitial densities), or pleural effusion as observed on chest radiographs (Cherian et al., 2005). Severity categorization adhered to the American guidelines for community-acquired pneumonia in children, with cases classified as severe based on ≥1 major or ≥2 minor criteria (Bradley et al., 2011). Hypoxia was defined as sustained saturation of peripheral oxygen or pulse oximetry measurement <92% on room air. The criteria for respiratory distress in children with pneumonia were as follows: (1) any of the following signs, including tachypnea (respiratory rate >60 breaths/min for these aged 0-2 months, >50 breaths/min for these aged 2-12 months, >40 breaths/min for these aged 1-5 years, and >20 breaths/min for these aged more than 5 years), dyspnea, retractions (suprasternal, intercostal, or subcostal), grunting, nasal flaring, apnea, altered mental status and pulse oximetry measurement <92% on room air (Bradley et al., 2011); (2) The ratio of the partial pressure of arterial oxygen to fraction of inspired oxygen <300 mmHg according to arterial blood gas analysis. Due to the study’s retrospective nature, written informed consent was waived as no personally identifiable information was utilized. Patients coinfected with human coronaviruses were excluded from statistical analysis, accounting for missing data.

### Statistical analysis

The Statistical Package for the Social Sciences (version 27.0) was employed for statistical analyses. Continuous variables are described as means ± standard deviation or medians (interquartile ranges), whereas categorical data are presented as frequencies and percentages. Differences between groups were assessed using chi-square or Fisher’s exact tests for categorical variables and t-tests or Mann-Whitney U tests for continuous variables.

## Results

### Demographic characteristics of hospitalized patients with HAdV-7 and HAdV-3 associated LRTIs

**Figure 1:**
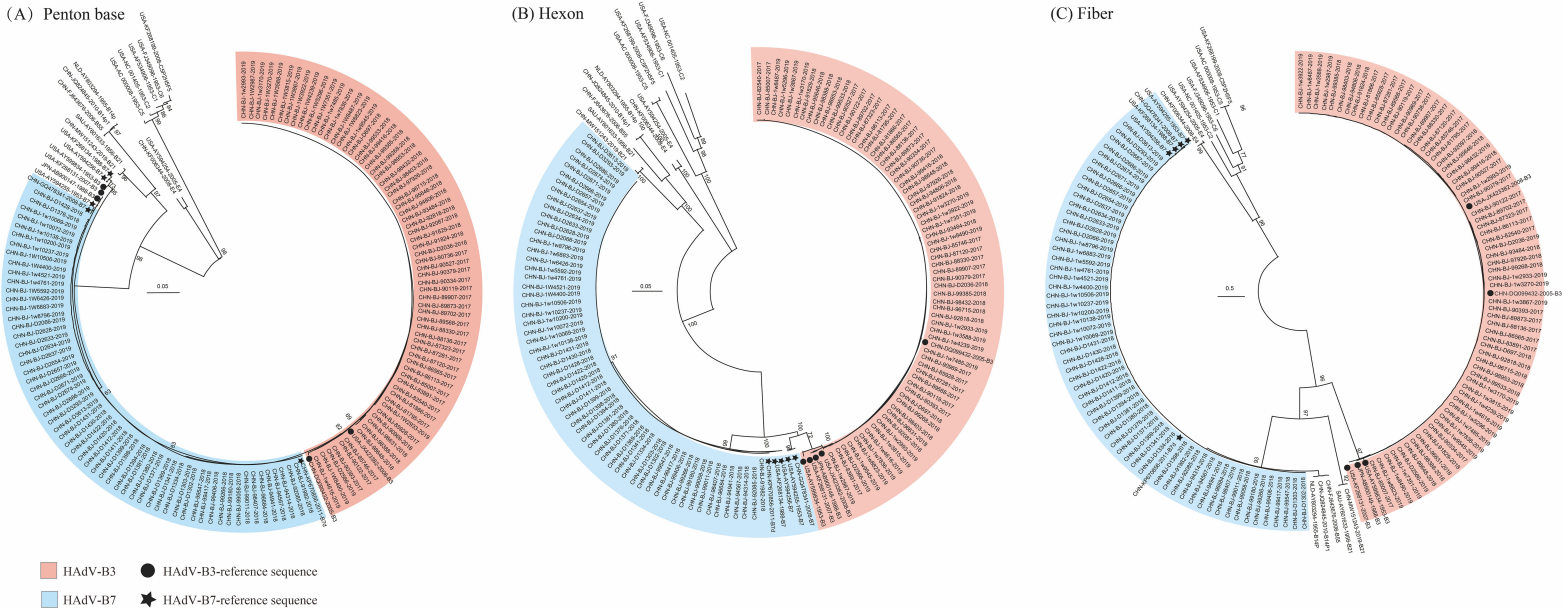
The phylogenetic trees of the penton base **(A)**, hexon **(B)**, and fiber genes **(C)** of the 126 identified HAdV-positive clinical specimens were generated using the General Time Reversible (GTR) model by MEGA X with the neighbor-joining method and 1,000 bootstrap replicates. Additional reference sequences were retrieved from GenBank for context and comparison.

Among 1311 respiratory specimens collected from children hospitalized with acute respiratory infection during the study period, 147 cases tested positive for HAdV (147/1311, 11.2%). Among specimens positive for HAdV, 126 clinical samples were successfully typed as HAdV-B3 and B7 by phylogenetic analyses of the penton base, hexon, and fiber genes (**Figure 1** **;** **Supplementary Table 1**). Of these, 67 cases were identified as HAdV-B3 (45.58%, 67/147) and 59 cases as HAdV-B7 (40.14%, 59/147). The phylogenetic analyses results were consistent with the BLAST results. Panels (A), (B), and (C) in **Figure 1** displayed the phylogenetic relationships of the penton base, hexon, and fiber genes, respectively. Ultimately, 66 cases of HAdV-3 and 58 of HAdV-7 associated LRTIs with detailed clinical information were enrolled.

As illustrated in **Table 1**, the median age of hospitalized patients with HAdV-7-associated LRTIs was 3.7 years (range: 0.3-9.7 years), whereas for HAdV-3-associated LRTIs, it was 3.3 years (range: 0.56-10.9 years). The demographic characteristics of patients infected with HAdV-3 and HAdV-7 were comparable, with no significant differences in age, gender distribution, or underlying medical conditions noted.

**Table 1:** Comparison of demographics, underlying medical conditions and clinical features in 124 hospitalized patients with HAdV-3 and HAdV-7 associated LRTIs.

Indicators	HAdV-7(n=58)	HAdV-3(n=66)	*p* value
Male	42 (72.4)	40 (60.6)	0.166
Median age, years (range)	3.3 (0.56-10.9)	3.7 (0.3-9.7)	0.154^§^
At least one underlying medical condition	12 (20.7)	15 (22.7)	0.784
Cardiac condition	8 (13.8)	8 (12.1)	0.782
Respiratory condition	5 (8.6)	6 (9.1)	0.927
Chronic neurological condition	2 (3.4)	3 (4.5)	1.000
Prematurity	1 (1.7)	2 (3.0)	1.000
immunodeficiency disorder	1 (1.7)	1 (1.5)	1.000
Systemic symptoms and signs			
Fever	58 (100)	59 (89.4)	0.014
Duration of fever (≥39°C), (days)	7.3 ± 4.8	5.1 ± 3.9	0.005
Fever (>39°C) for more than 5 days	33 (56.9)	26 (39.4)	0.052
Altered mental status	21 (36.2)	15 (22.7)	0.099
Chills	8 (13.8)	10 (15.2)	0.830
Malaise	2 (3.4)	5 (7.6)	0.447
Respiratory symptoms and signs			
Cough	58 (100)	57 (86.4)	0.003
Inflamed throat	55 (94.8)	52 (78.8)	0.010
Moist rales	51 (87.9)	47 (71.2)	0.022
Dyspnea or wheezing	19 (32.8)	17 (25.8)	0.391
Rhinorrhea	14 (24.1)	7 (10.6)	0.045
Nasal obstruction	5 (8.6)	8 (12.1)	0.526
Sore throat	3 (5.3)	3 (4.5)	1.000
Extrapulmonary symptoms and signs			
Decreased appetite	32 (55.2)	10 (15.2)	<0.001
Diarrhea	10 (17.2)	3 (4.5)	0.021
Abdominal pain	7 (12.1)	0	0.004
Vomiting	6 (10.3)	2 (3.0)	0.145
Altered or loss of consciousness	2 (3.4)	3 (4.5)	1.000
Treatment			
Interferon	56 (96.6)	54 (81.8)	0.010
Thymopentin	44 (75.9)	47 (71.2)	0.559
Immunoglobulin	43 (74.1)	32 (48.5)	0.004
Glucocorticoids	32 (55.2)	26 (39.4)	0.079
Outcome			
Course of disease upon hospitalization, (days)	6.9 ± 4.1	13.5 ± 10.0	<0.001
Length of hospital stay, (days)	11.6 ± 7.5	9.1 ± 10.9	0.140
Pneumonia	58 (100)	59 (89.4)	0.014
Severe pneumonia	25 (43.1)	18 (27.3)	0.065
Hypoxia	20 (34.5)	8 (12.1)	0.003
Respiratory failure	10 (17.2)	6 (9.1)	0.177
Mechanical ventilation	2 (3.4)	6 (9.1)	0.281
Intensive care unit admission	2 (3.4)	4 (6.1)	0.684
Death	0	1 (1.5)	1.000

Values are no. (%) of patients or means ± standard deviation, unless otherwise indicated.

^§^Differences between groups were analyzed using Mann-Whitney U tests for continuous variables of non-normal distribution.

### Comparison of clinical severity in hospitalized patients with HAdV-7 and HAdV-3 associated LRTIs

Compared to those with HAdV-3 infections, patients with HAdV-7 were significantly more likely to experience an increased incidence of symptoms such as prolonged fever (100% vs 89.4%, *p*=0.014), cough (100% vs 86.4%, *p*=0.003), inflamed throat (94.8% vs 78.8%, *p*=0.010), moist rales (87.9% vs 71.2%, *p*=0.022), decreased appetite (55.2% vs 15.2%, *p*<0.001), rhinorrhea (24.1% vs 10.6%, *p*=0.045), diarrhea (17.2% vs 4.5%, *p*=0.021), and abdominal pain (12.1% vs 0, *p*=0.004) (**Table 1**). Pediatric patients infected with HAdV-7 experienced significantly longer fever durations exceeding 39°C than those with HAdV-3 infections (7.3 ± 4.8 vs 5.1 ± 3.9 days, *p*=0.005). The mean disease course upon hospitalization for patients with HAdV-7 was 6.9 ± 4.1 days, considerably shorter than that of the HAdV-3 group (13.5 ± 10.0 days, *p*<0.001). Both groups presented comparable rates of dyspnea or wheezing.

In terms of treatment and outcomes, patients with HAdV-7 infections notably required more frequent medications, such as interferon (96.6% vs 81.8%, *p*=0.010) and immunoglobulin (74.1% vs 48.5%, *p*=0.004), and demonstrated higher morbidity rates of pneumonia (100% vs 89.4%, *p*=0.014) and hypoxia (34.5% vs 12.1%, *p*=0.003) than those with HAdV-3 infections (**Table 1**). Despite a higher proportion of severe pneumonia diagnoses (43.1% vs 27.3%), glucocorticoids use (55.2% vs 39.4%) and occurrence of respiratory failure (17.2% vs 9.1%) among HAdV-7 patients, statistical significance was not reached. Unexpectedly, admission to the intensive care unit (3.4% vs 6.1%) and the use of mechanical ventilation (3.4% vs 9.1%) were more frequent in the HAdV-3 group, among which one child died due to complications.

Laboratory findings for patients infected with HAdV-7 differed significantly from those infected with HAdV-3, showing a greater likelihood of leukopenia (21.1% vs 3.1%, *p*=0.002), neutropenia (29.8% vs 14.1%, *p*=0.035), markers indicative of liver damage such as alanine aminotransferase >80 U/L (10.5% vs 1.6%, *p*=0.051), aspartate aminotransferase >80 U/L (21.1% vs 4.7%, *p*=0.006), lactate dehydrogenase >300 U/L (78.9% vs 59.4%, *p*=0.021), α1-hydroxybutyrate dehydrogenase >260 U/L (63.2% vs 43.8%, *p*=0.033), coagulation disorders such as D-Dimer>1.65 mg/L (64.9% vs 12.5%, *p*<0.001), fibrinogen degradation products [6.2 (3.0-12.2) vs 3.0 (2.3-5.1)g/L, *p*=0.002], as well as less likelihood of marker indicative of myocardial damage such as creatine kinase-MB >6.33 ng/ml (3.5% vs 14.1%, *p*=0.044) (**Table 2**). Despite these differences, no significant disparities were found in other common blood parameters.

**Table 2:** Comparison of laboratory findings in 121 hospitalized patients with HAdV-3 and HAdV-7 associated with LRTIs.

Laboratory parameters	HAdV-7 (n=57)	HAdV-3 (n=64)	*p* value
Blood cell counts (×10^9^ cells/L)			
White blood cells	9.4 ± 9.4	10.8 ± 5.7	0.310
White blood cells >15×10^9^ cells/L	5 (8.8)	11 (17.2)	0.192
White blood cells <4.0×10^9^ cells/L	12 (21.1)	2 (3.1)	0.002
Neutrophils	5.4 ± 9.1	6.0 ± 4.9	0.660
Neutrophils<1.5×10^9^ cells/L	17 (29.8)	9 (14.1)	0.035
Platelets, median (IQR)	292 (172.0-432.5)	310 (241.5-411.5)	0.315^§^
Hemoglobin (g/L)	110.4 ± 12.6	114.0 ± 11.4	0.105
C-reactive protein (mg/L)	23.0 ± 26.8	27.3 ± 41.3	0.501
Procalcitonin (ng/ml)	1.0 ± 1.7	0.9 ± 1.8	0.784
Alanine aminotransferase (U/L)	35.3 ± 29.8	21.7 ± 35.0	0.024
Alanine aminotransferase >80 U/L	6 (10.5)	1 (1.6)	0.051
Aspartate aminotransferase (U/L), median (IQR)	39.7 (29.9-71.9)	32.7 (25.9-48.0)	0.061^§^
Aspartate aminotransferase >80 U/L	12 (21.1)	3 (4.7)	0.006
Lactate dehydrogenase (U/L), median (IQR)	434.0 (324.0-734.5)	322.0 (271.0-412.8)	<0.001^§^
Lactate dehydrogenase >300U/L	45 (78.9)	38 (59.4)	0.021
α1 hydroxybutyrate dehydrogenase (U/L), median (IQR)	314.0 (238.5-542.0)	248.0 (203.3-338.0)	<0.001^§^
α1 hydroxybutyrate dehydrogenase >260 U/L	36 (63.2)	28 (43.8)	0.033
Creatine kinase (U/L)	141.9 ± 282.3	100.9 ± 133.7	0.302
Creatine kinase >220U/L	5 (8.8)	4 (6.3)	0.733
Creatine kinase-MB (ng/ml), median (IQR)	0.6 (0.2-1.3)	0.7 (0.2-1.8)	0.007^§^
Creatine kinase-MB >6.33ng/ml	2 (3.5)	9 (14.1)	0.044
D-Dimer (mg/L), median (IQR)	2.6 (0.6-5.0)	0.4 (0.3-0.9)	<0.001^§^
D-Dimer>1.65mg/L	37 (64.9)	8 (12.5)	<0.001
Fibrinogen degradation products (g/L), median (IQR)	6.2 (3.0-12.2)	3.0 (2.3-5.1)	0.002^§^
Prothrombin time (s)	11.0 ± 1.1	11.3 ± 2.1	0.325
Activated partial thromboplastin time (s)	34.2 ± 6.1	31.7 ± 5.5	0.018
Thrombin time (s), median (IQR)	18.0 (16.2-19.5)	16.4 (15.3-18.3)	0.004^§^
Urea (mmol/L)	3.4 ± 1.2	3.1 ± 1.0	0.132
Creatinine (µmol/L), median (IQR)	28.6 (22.5-36.6)	26.1 (22.0-30.6)	0.042^§^

Values are no. (%) of patients or means ± standard deviation, unless otherwise indicated. IQR: interquartile range.

^§^Differences between groups were analyzed using Mann-Whitney U tests for continuous variables of non-normal distribution.

Imaging of chest X-ray or computed tomography scan revealed a spectrum of radiological abnormalities (**Table 3**). Imaging studies revealed that HAdV-7 patients had a higher frequency of alveolar or interstitial infiltrates (98.3% vs 85.5%, *p*=0.017), consolidation (50.0% vs 27.4%, *p*=0.011) and pleural effusion (32.8% vs 6.5%, *p*<0.001) than those with HAdV-3. Unexpectedly, multilobe infiltrates were slightly more common in HAdV-3-infected patients (32.8% vs 43.5%, *p*=0.224). Both groups exhibited similar rates of atelectasis and small airway disease, with two HAdV-7-infected patients experiencing severe complications like pulmonary necrosis and emphysema.

**Table 3:** Comparison of radiological findings in 120 hospitalized children of HAdV-3 and HAdV-7 associated LRTIs.

Radiological findings	HAdV-7(n=58)	HAdV-3(n=62)	*p* value
Alveolar or interstitial infiltrate	57 (98.3)	53 (85.5)	0.017
Consolidation	29 (50.0)	17 (27.4)	0.011
Multilabor infiltrates (≥3 labors)	19 (32.8)	27 (43.5)	0.224
Pleural effusion	19 (32.8)	4 (6.5)	<0.001
Atelectasis	5 (8.6)	6 (9.7)	0.841
Small airway disease	5 (8.6)	3 (4.8)	0.481
Thickening of subsegmental bronchi wall	5 (8.6)	1 (1.6)	0.078
Pleural thickening or exudation	4 (6.9)	4 (6.5)	1.000
Pneumothorax or mediastinal emphysema	2 (3.4)	0	0.232
Pulmonary necrosis	2 (3.4)	0	0.232

Values are no. (%) of patients.

### Comparison of co-infections in hospitalized patients with HAdV-7 and HAdV-3 associated LRTIs

Among the HAdV-7 positive patients (n=35), 33 (89.5%) exhibited co-infections with other respiratory pathogens, compared to an 82.6% co-infection rate among the HAdV-3 positive patients (n=46). Patients infected with HAdV-3 demonstrated a higher propensity for dual infections (29.6% vs 41.3%, *p*=0.081; **Supplementary Table 2**). Conversely, patients infected with HAdV-7 exhibited a greater likelihood of multiple infections (56.8% vs 41.3%, *p*=0.007; **Supplementary Table 3**). Patients with HAdV-7 infection demonstrated significantly higher rates of co-infection with other respiratory viruses (48.6% vs 26.1%, *p*=0.035), atypical pathogens (80.0% vs 32.6%, *p*<0.001), and marginally higher rates with respiratory bacteria (62.9% vs 56.5%) compared to those with HAdV-3 infection (**Table 4**). Significantly higher frequencies of MP and parainfluenza virus co-infection were observed in patients with HAdV-7 compared to those with HAdV-3 (77.1% vs 32.6%, *p*<0.001; 20.0% vs 2.2%, *p*=0.018, respectively). Furthermore, HAdV-7 positive children were frequently co-infected with pathogens such as *Staphylococcus aureus*, respiratory syncytial virus, bocavirus, rhinovirus, Candida albicans or Aspergillus fumigatus, whereas HAdV-3 positive cases showed a higher propensity for co-infection with *Hemophilus influenzae*, *Methicillin resistant staphylococcus*, *Acinetobacter baumannii*, and exhibited higher positive sputum or BALF cultures, albeit these were not statistically significantly different. Co-infection rates with *Streptococcus pneumoniae* were similar between the two groups.

**Table 4:** Comparison of co-detections in 81 hospitalized children of etiologic HAdV-3 and HAdV-7 associated LRTIs.

Pathogens	HAdV-7(n=35)	HAdV-3(n=46)	P value
At least one other respiratory pathogen	33 (89.5)	38 (82.6)	0.114
1 other pathogen (dual infection)	8 (29.6)	19 (41.3)	0.081
≥2 other pathogens (multiple infections)	25 (56.8)	19 (41.3)	0.007
Respiratory other viruses	17 (48.6)	12 (26.1)	0.037
Parainfluenza virus	7 (20.0)	1 (2.2)	0.018
Respiratory syncytial virus	6 (17.1)	6 (13.0)	0.607
Bocavirus	3 (8.6)	1 (2.2)	0.311
Rhinovirus	3 (8.6)	1 (2.2)	0.311
Influenza A	0	3 (6.5)	0.255
Influenza B	0	2 (4.3)	0.503
Metapneumovirus or Coronavirus	0	0	
Respiratory bacteria	22 (62.9)	26 (56.5)	0.565
*Haemophilus influenzae*	5 (14.3)	13 (28.3)	0.134
*Streptococcus pneumoniae*	7 (20.0)	9 (19.6)	0.961
*Staphylococcus aureus*	8 (22.9)	4 (8.7)	0.076
*Methicillin resistant staphylococcus*	3 (8.6)	5 (10.9)	1.000
*Acinetobacter baumannii*	1 (2.9)	3 (6.5)	0.630
*Escherichia coli*	0	1 (2.2)	1.000
*Klebsiella pneumoniae*	0	2 (4.3)	0.503
*Pseudomonas aeruginosa*	0	1 (2.2)	1.000
*Stenotrophomonas maltophilia*	0	1 (2.2)	1.000
Others^†^	1 (2.9)	4 (8.7)	0.383
Respiratory atypical pathogens	28 (80.0)	32.6 (32.6)	<0.001
*Mycoplasma pneumoniae*	27 (77.1)	15 (32.6)	<0.001
*Chlamydia pneumoniae*	1 (2.9)	0	0.432
*Legionella pneumophila*	0	0	
*Aspergillus fumigatus* or *Candida albicans*	2 (5.7)	1 (2.2)	0.575
Positive sputum or bronchoalveolar lavage fluid culture	3 (8.6)	11 (23.9)	0.070

Values are no. (%) of patients.

^†^other pathogens indicated bacteria including Enterobacter cloacae, Enterobacter aerogen, Burkholderia cepacian and Streptococcus dysgalactiae, Moraxella catarrhalis and mycobacterium tuberculosis complex.

### Comparison of immune response in hospitalized patients with HAdV-7 and HAdV-3 associated LRTIs

**Figure 2:**
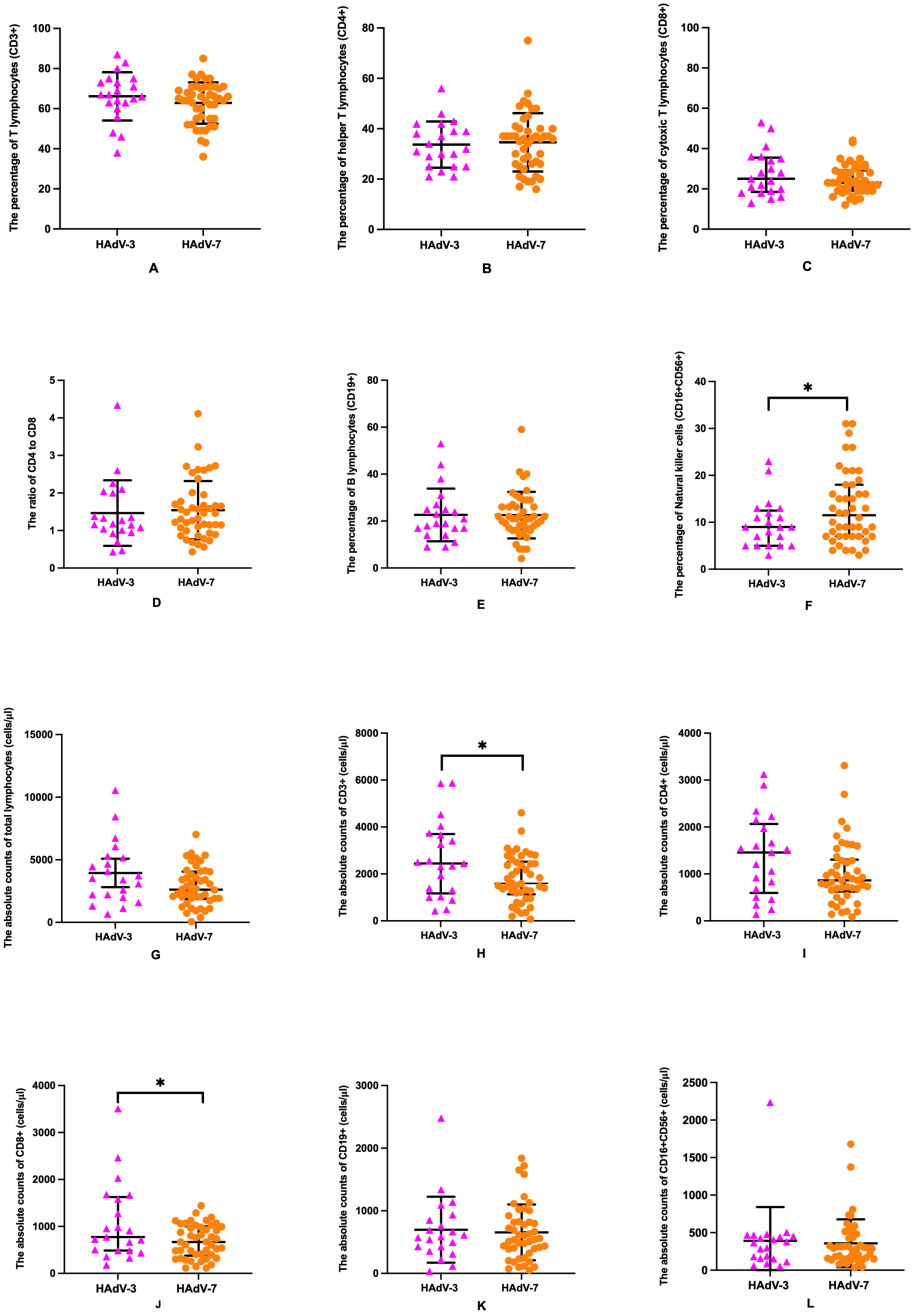
Comparative analysis of lymphocyte subsets in HAdV-3 (n=21) and HAdV-7 (n=46) infected patients with LRTIs. Percentages of CD3+ T lymphocytes **(A)** and CD8+ T lymphocytes **(C)**, percentages and absolute counts of CD4+ T lymphocytes **(B, I)**, CD19+ B lymphocytes **(E, K)**, absolute counts of total lymphocytes **(G)** and CD16+CD56+ natural killer cells **(L)**, and the ratio of CD4+ to CD8+ T lymphocytes **(D)** were shown no significant difference between HAdV-3 group and HAdV-7 group. Percentage of CD16+CD56+ natural killer cells **(F)** was with a significant increase in the HAdV-7 group, while absolute counts of CD3+ T lymphocytes **(H)** and CD8+ T lymphocytes **(J)** were with a significant reduction in the HAdV-7 group. Error bars represented the mean with standard deviation or median interquartile range. *: Statistically significant differences were indicated between two groups (p<0.05).

The effects of HAdV-7 and HAdV-3 infections on major lymphocyte subset alterations, immunoglobulins and complement levels in peripheral blood were investigated among 67 children with LRTIs (**Table 5**, **Figure 2**). Patients with HAdV-7 infection exhibited a decrease in total lymphocyte counts in peripheral blood compared to those with HAdV-3 infection, though this difference was not statistically significant [2612.0 (1873.2-4057.3) vs 3528.1 (2104.1-5228.5) cells/l, *p*=0.080]. Among T cell subsets, HAdV-7-infected patients showed significant decreases in CD3+ and CD8+ T cell counts [1596.8 (1132.5-2521.9) vs 2444.8 (1171.0-3700.8) cells/l, *p*=0.042; 668.6 (378.3-1000.8) vs 774.0 (485.9-1624.5) cells/l, *p*=0.045, respectively] compared to those with HAdV-3 infection, suggesting an imbalance in T cell subsets and potential immune dysregulation. HAdV-7 positive patients had lower CD4+ T cell counts [861.6 (626.6-1305.6) vs 1456.0 (595.0-2064.2) cells/l], CD19+ B cell frequencies (654.7 ± 446.0 vs 711.9 ± 511.4 cells/l), and CD16+CD56+ NK cell numbers (358.2 ± 318.6 vs 390.5 ± 450.5 cells/l) than those with HAdV-3 infection, though these differences were not statistically significant. The HAdV-7 positive group exhibited a higher percentage of CD16+CD56+ NK cells compared to the HAdV-3 positive group [11.5% (7.0%-18.0%) vs 9.0% (5.0%-12.5%), *p*=0.044], with comparable percentages of other lymphocyte subsets between the groups. No significant differences were noted in the levels of complement 3 and 4, or immunoglobulins G, A, M, and E between patients infected with HAdV-7 and those with HAdV-3.

**Table 5:** Comparison of immunological features in 67 hospitalized children of HAdV-7 and HAdV-3 associated LRTIs.

Immunological features	HAdV-7(n=46)	HAdV-3(n=21)	*p* value
Total lymphocytes (cells/µl), median (IQR)	2612.0 (1873.2-4057.3)	3528.1 (2104.1-5228.5)	0.080^§^
T lymphocytes (CD3+, cells/µl), median (IQR)	1596.8 (1132.5-2521.9)	2444.8 (1171.0-3700.8)	0.042^§^
Helper T lymphocytes (CD4+, cells/µl), median (IQR)	861.6 (626.6-1305.6)	1456.0 (595.0-2064.2)	0.088^§^
Cytoxic T lymphocytes (CD8+, cells/µl), median (IQR)	668.6 (378.3-1000.8)	774.0 (485.9-1624.5)	0.045^§^
B lymphocytes (CD19+, cells/µl)	654.7 ± 446.0	711.9 ± 511.4	0.643
Natural killer cells (CD16+CD56+, cells/µl)	358.2 ± 318.6	390.5 ± 450.5	0.737
T lymphocytes (CD3+, %)	62.8 ± 10.3	66.1 ± 12.0	0.248
Helper T lymphocytes (CD4+, %)	34.6 ± 11.6	33.7 ± 9.2	0.756
Cytoxic T lymphocytes (CD8+, %), median (IQR)	23.0 (19.0-29.3)	25.0 (18.5-35.5)	0.254^§^
CD4/CD8	1.5 ± 0.8	1.5 ± 0.9	0.719
B lymphocytes (CD19+, %)	22.5 ± 9.9	22.6 ± 11.2	0.972
Natural killer cells (CD16+CD56+, %), median (IQR)	11.5 (7.0-18.0)	9.0 (5.0-12.5)	0.044^§^
Complement 3 (g/L), median (IQR)	1.0 (0.8-1.2)	1.1 (0.9-1.2)	0.645^§^
Complement 4 (g/L)	0.4 ± 0.6	0.3 ± 0.1	0.340
Immuglobulin G (g/L)	12.4 ± 14.9	8.5 ± 4.5	0.249
Immuglobulin A (g/L)	0.9 ± 0.6	0.8 ± 0.7	0.478
Immuglobulin M (g/L), median (IQR)	1.5 (1.1-2.4)	0.9 (0.7-1.7)	0.997^§^
Immuglobulin E (IU/ml), median (IQR)	75.9 (17.3-195.3)	37.8 (15.9-299.5)	0.372^§^

Data are shown as means ± standard deviation, medians (range) or number (%).

Values are means ± standard deviation, unless otherwise indicated.

IQR: interquartile range.

^§^Differences between groups were analyzed using Mann-Whitney U tests for continuous variables of non-normal distribution.

## Discussion

The current analysis highlights significant differences in clinical severity and outcomes between HAdV-7 and HAdV-3 infections, with HAdV-7 patients exhibiting a higher incidence of severe symptoms, including prolonged fever, pneumonia, hypoxia, liver damage, and coagulation disorders. Radiological findings further support the clinical observation of a more severe disease in HAdV-7 infections, evidenced by a higher frequency of alveolar or interstitial infiltrates, consolidation, and pleural effusion compared to those in HAdV-3 infections. This is consistent with the understanding that HAdV-7 can lead to more extensive lung involvement and damage, potentially explaining the increased severity and adverse clinical outcomes observed in these patients. Studies have demonstrated that HAdV-7 can lead to more severe respiratory illnesses, often characterized by higher mortality and requiring increased mechanical ventilation and intensive care unit admissions compared to HAdV-3 (Lin et al., 2017; Dai et al., 2020; Wang et al., 2023). Previous studies have indicated that HAdV-7 exhibits more robust replication and triggers an exacerbated cytokine response compared to HAdV-3, resulting in more severe airway inflammation (Fu et al., 2019; Siew et al., 2020). In the current study, higher frequencies of mechanical ventilation and intensive care unit admissions were observed in the HAdV-3 group; however, mortality remained low across both groups. The investigation in current study revealed a higher incidence of immunoglobulin and glucocorticoid use in treating individuals with HAdV-7 compared to those infected with HAdV-3. Intravenous immunoglobulin and/or glucocorticoid treatments are effective in preventing severe adenovirus pneumonia in children (Oliveira et al., 2020; Cai et al., 2023). Corroborating these observations in this study, Li L et al. (Li et al., 2018) demonstrated that strategic glucocorticoid and immunoglobulin use in adenovirus pneumonia treatment effectively reduces complication risks and enhances recovery prospects.

In this study, co-infection rates were notably higher in patients testing positive for HAdV-7 compared to those with HAdV-3. Furthermore, the current analysis indicated a greater propensity for patients infected with HAdV-3 to present with dual infections, whereas those with HAdV-7 infections were more commonly associated with multiple infections. This discrepancy suggests that HAdV-7 may predispose children to a broader spectrum of secondary infections, potentially owing to differences in pathogenic mechanisms and immune evasion strategies between these adenovirus serotypes. A notable aspect of this investigation is the remarkably high detection rate of MP infection in subjects infected with either HAdV-7 (77.1%) or HAdV-3 (32.6%), identifying MP as a predominant co-infecting agent with adenovirus. These findings underscore the importance of considering HAdV-MP co-infections in the initial empirical treatment strategy for pediatric severe community-acquired pneumonia, aiding in the prognosis of HAdV-infected patients. Previous investigations of Chinese children with LRTIs have noted HAdV-MP co-infection rates ranging from 10% to 16% among all children infected with adenovirus (Lu et al., 2013; Chen et al., 2016). A surge to 58% in co-infection rates has been documented in a recent study by Chen Q et al (Chen et al., 2024). The elevated detection rates of HAdV-7-MP co-infections could stem from the selection of more severe cases, an outbreak of MP and HAdV-7 during the study period enhanced sensitivity of molecular assays (Yang et al., 2021; Cheng et al., 2022), and the prolonged presence of MP nucleic acid in the airway (Liu et al., 2019). These findings in current study align with recent literatures that suggests HAdV-MP co-infections significantly impact the clinical severity and outcomes of patients with LRTIs. Individuals with concurrent HAdV and MP infections exhibit prolonged febrile responses and more acute illness than those afflicted by MP alone (Gao et al., 2020; Zhou et al., 2020; Zhang et al., 2021; Zhou et al., 2022; Chen et al., 2024). Co-infection with MP typically results in prolonged fever, indicating that co-infections may delay pathogen elimination and intensify the host’s immune response due to increased internal and external pyrogens (Gao et al., 2020). Co-infected cases of HAdV-MP exhibited more frequent occurrences of lung consolidation, atelectasis, pleural effusion, and multiple lung lobe lesions, indicating that co-infections can exacerbate pulmonary inflammation in children with adenovirus pneumonia through direct damage or indirect immune responses (Wei et al., 2022). HAdV-7 may prolong pathogen clearance duration and exacerbate the host’s immune response, resulting in prolonged inflammation (Chen et al., 2021). Although the precise mechanisms remain unclear, a potential synergistic interaction between MP and other pathogens is suggested (Cimolai et al., 1995). A study analyzing lung microbiota in BALF revealed that HAdV-MP co-infections increase the diversity within groups while maintaining similar species richness, offering insights into potential mechanisms (Zhou et al., 2022). Future research investigating the mechanisms underlying these observations and their implications for treatment strategies is essential. Addressing the challenges posed by co-infections necessitates a multifaceted approach that combines robust diagnostic strategies, comprehensive treatment regimens, and preventive measures to mitigate the burden of HAdV-associated respiratory diseases in children.

This study highlights the differential impact of HAdV-7 and HAdV-3 infections on the immune system’s cellular components in pediatric patients with LRTIs. Comparative analysis between HAdV-7 and HAdV-3 infected patients revealed a notable, albeit not statistically significant, decrease in total lymphocyte counts in the peripheral blood of HAdV-7 infections. The observed reduction in T cell frequencies in the bloodstream may stem from the demise of T cells engaged in combating HAdV-7 or from virus-induced inhibition of T cell production. This observation supports the hypothesis that viral mechanisms, such as direct lymphocyte destruction, impaired proliferation, or migration to infection sites, contribute to the observed lymphocytopenia (Sun et al., 2023). Regarding T cell subsets, current findings indicate a significant decrease in both CD3+ T cells and CD8+ cytotoxic T cells, as well as marginal decrease in CD4+ helper T cells among HAdV-7 infected patients compared to those with HAdV-3 infections. This disparity suggests a pronounced impact of HAdV-7 on the T cell compartment, potentially leading to an imbalance in T cell subsets and ensuing immune dysregulation. This might reflect a distinct immune response strategy against different adenovirus serotypes and suggest that the primary distinctions in immune response are confined to aspects of cellular immunity. These findings are supported by a broader understanding of adenovirus-specific T cell responses in humans, as detailed by Hutnick NA et al (Hutnick et al., 2010). Adenovirus-specific T cells, both CD4+ and CD8+, exhibit a wide range of functionality and can respond to various adenovirus serotypes due to cross-reactivity against conserved hexon regions. Sun J et al. (Sun et al., 2023) reported a significant decline in the percentages of CD3+ T cells and NK cells in patients with HAdV-7 infection, categorized into upper respiratory infection, common pneumonia, and severe pneumonia groups, with the decline being more pronounced in NK cells and CD4+ T cells, but not in CD8+ T cells. Chen WW et al. (Chen et al., 2014) found that patients with severe HAdV-55 infections exhibited significantly elevated levels of IL-17+CD4+ cells and reduced levels of IL-17+CD8+ cells compared to those with asymptomatic HAdV-55 infections. The notable reduction in T cell subsets, accompanied by increased NK cell percentages in HAdV-7 infections, may have implications for understanding the pathogenesis of these infections and developing targeted therapeutic strategies. Future research should focus on further elucidating the mechanisms behind these observations, potentially exploring the role of adenovirus-specific T cell cross-reactivity in shaping the immune response to different adenovirus infections.

To better understanding the molecular and immunological mechanisms underlying the differences between HAdV-7 and HAdV-3, the following research directions are proposed. First,

further investigation into the viral factors and host cell receptors involved in the differential immune response to these two adenoviruses could provide valuable insights. A comparative study of viral proteins and their interactions with immune cells may reveal key determinants of immune modulation. Second, a comprehensive exploration of the cytokine and chemokine profiles associated with HAdV-7 and HAdV-3 infections is essential, particularly in relation to their roles in driving inflammatory responses and exacerbating disease severity. Such studies will be crucial for elucidating the divergent clinical outcomes. Additionally, leveraging genomic and proteomic approaches to identify host genetic factors and protein interactions that contribute to the severity of HAdV-7 infections will be important. Finally, the development and use of animal models to investigate HAdV-7 and HAdV-3 infections will enable a more detailed examination of *in vivo* immune responses and the pathophysiological mechanisms contributing to the observed differences.

This study has several limitations. First, its retrospective nature. Second, the relatively small sample size may not fully represent the broader population affected by HAdV-3 and HAdV-7 associated LRTIs. Third, the inability to detect viral load and the presence of HAdV in blood samples limits understanding of the virus’s role in clinical pathogenicity. Particularly in cases of co-infection with other respiratory viruses, the precise contribution of HAdV to disease severity remains unclear. These limitations indicate the need for multicenter, longitudinal studies with larger and more diverse populations to confirm and expand upon these findings.

In conclusion, hospitalized children with HAdV-7-associated LRTIs exhibit greater severity, multiple infections, higher incidence of co-infections with MP, and significant potential for greater cellular immune dysregulation compared to those with HAdV-3 infection, indicating a more severe clinical course and distinct pathogenic profiles. These findings underscore the importance of continuous targeted surveillance, accurate diagnosis, innovative therapeutic interventions, and tailored clinical management strategies to mitigate the impact of these infections on pediatric populations, highlighting the potential for vaccine development to combat the significant health burden posed by HAdV infections. Future research should focus on elucidating the molecular mechanisms underlying the pathogenicity differences between HAdV serotypes and exploring targeted therapeutic interventions to enhance patient outcomes.

## Data Availability

The datasets presented in this study can be found in online repositories. The names of the repository/repositories and accession number(s) can be found in the article/**Supplementary Material**.
