# Supplementary material for: Impact of co-infections and immune responses on clinical severity of human adenovirus 3 and 7 infections in hospitalized children with lower respiratory tract infections: a comparative study
Source: Front Cell Infect Microbiol. 2025 Jan 9;14:1482787. doi: 10.3389/fcimb.2024.1482787 (PMC11754186; doi:10.3389/fcimb.2024.1482787)
Supplement: Supplementary file 1 [file Table1.docx]

**Supplementary Table 1:** List of reference HAdV strains used in the phylogenetic analyses.

| GenBank ID | Organism | Country of origin | Collection date | Strain Name |
| --- | --- | --- | --- | --- |
| AY599834 | Human adenovirus B3 | USA | 1953 | Strain GB |
| JX423382 | Human adenovirus B3 | USA | 2008 | ak34_AdV3a2 |
| DQ099432 | Human adenovirus B3 | CHN | 2005 | Guangzhou01 |
| AB900147/148/149 | Human adenovirus B3 | JPN | 1988 | F1_Adv3a52 |
| KF268131 | Human adenovirus B3 | USA | 2007 | UFL_Adv3a17 |
| AY594255 | Human adenovirus B7 | USA | 1953 | Gomen |
| KP670856 | Human adenovirus B7d | CHN | 2011 | GZ6965 |
| AY594256 | Human adenovirus B7 | USA | - | Vaccine strain |
| GQ478341 | Human adenovirus B7 | CHN | 2008 | GZ08 |
| KF268134 | Human adenovirus B7 | USA | 1988 | CL_43 |
| AY803294 | Human adenovirus B14p | NLD | 1955 | de_Wit |
| JQ824845 | Human adenovirus B14p1 | CHN | 2010 | CHN/GZ01 |
| FJ643676 | Human adenovirus B55 | CHN | 2006 | QS-DLL |
| MW151243 | Human adenovirus B21 | CHN | 2019 | GZ09107 |
| AY601633 | Human adenovirus B21 | SAU | 1956 | AV-1645 |
| AF534906 | Human adenovirus C1 | USA | 1953 | HAdV-1 |
| NC_001405 | Human adenovirus C2 | USA | 1953 | HAdV-2 |
| AC_000008 | Human adenovirus C5 | USA | 1953 | HAdV-5 |
| KF268199 | Human adenovirus C5 | USA | 2008 | UFL_Adv5 |
| FJ349096 | Human adenovirus C6 | USA | 1953 | HAdV-6 |
| KF006344 | Human adenovirus E4 | CHN | 2008 | GZ01 |
| AY594254 | Human adenovirus E4 | USA | - | Vaccine strain |

**Supplementary Table 2**: Co-detection of HAdV-3 with other respiratory pathogens.

| 2 pathogens (n=19) | | 3 pathogens (n=11) | | ≥4 pathogens (n=8) | |
| --- | --- | --- | --- | --- | --- |
| Etiologic agents (HAdV-3 +) | n | Etiologic agents (HAdV-3 +) | n | Etiologic agents  (HAdV-3 +) | n |
| MP | 8 | SP+SA | 2 | HI+SP+MP | 1 |
| HI | 5 | SP+FluA | 1 | HI+EC +MRS | 1 |
| MRS | 2 | SP+HI | 1 | MP+RSV+FluB | 1 |
| SP | 2 | MP+SP | 1 | SP+RSV+EA | 1 |
| RSV | 1 | MP+HI | 1 | HI+SP+MP+RSV | 1 |
| CA | 1 | MP+MRV | 1 | HI+SA+AB+PA+ FluA | 1 |
|  |  | MP+FluA | 1 | KP+SM+BC+HRV+HBoV | 1 |
|  |  | HI+RSV | 1 | SA+MRS+AB+KP+FluB+ CA | 1 |
|  |  | HPIV+AB | 1 |  |  |
|  |  | RSV+CA | 1 |  |  |

Abbreviations: AB: *Acinetobacter baumannii*, BC: *Burkholderia cepacian*, CA: *Candida albicans*, EA: *Enterobacter aerogen*, EC: *Escherichia coli*, FluA/B: Influenza A/B, HBoV: human bocavirus, HI: *Haemophilus influenzae*, HPIV: human parainfluenza virus, HRV: human rhinovirus, KP: *Klebsiella pneumoniae*, MP: *Mycoplasma pneumoniae*, MRS: *Methicillin resistant staphylococcus*, PA: *Pseudomonas aeruginosa*, RSV: respiratory syncytial virus, SA: *Staphylococcus aureus*, SM: *Stenotrophomonas maltophilia*, SP: *Streptococcus pneumoniae*.

**Supplementary Table 3**: Co-detection of HAdV-7 with other respiratory pathogens.

| 2 pathogens (n=7) | | 3 pathogens (n=13) | | ≥4 pathogens (n=12) | |
| --- | --- | --- | --- | --- | --- |
| Etiologic agents (HAdV-7 +) | n | Etiologic agents (HAdV-7 +) | n | Etiologic agents  (HAdV-7 +) | n |
| MP | 4 | MP+HPIV | 4 | MP+SP+RSV | 1 |
| HBoV | 1 | MP+SP | 3 | MP+SA+MRS | 2 |
| HI | 1 | MP+RSV | 2 | MP+SA+RSV | 1 |
| SA | 1 | MP+HI | 1 | MP+SA+AB | 1 |
|  |  | MP+SA | 1 | SA+HPIV+HBoV | 1 |
|  |  | MP+HRV | 1 | MP+HI+HBoV | 1 |
|  |  | HPIV+HI | 1 | MP+SP+MRS | 1 |
|  |  |  |  | MP+SP+MRS+HRV | 1 |
|  |  |  |  | MP+SP+RSV+HPIV | 1 |
|  |  |  |  | CP+HI+RSV+CA | 1 |
|  |  |  |  | MP+EBC+HRV+CA+AF | 1 |

Abbreviations: AB: *Acinetobacter baumannii*, AF: *Aspergillus fumigatus*, CA: *Candida albicans*, CP: *Chlamydia pneumoniae*, EBC: *Enterobacter cloacae*, HBoV: human bocavirus, HI: *Haemophilus influenzae*, HPIV: human parainfluenza virus, HRV: human rhinovirus, MP: *Mycoplasma pneumoniae*, MRS: *Methicillin resistant staphylococcus*, RSV: respiratory syncytial virus, SA: *Staphylococcus aureus*, SP: *Streptococcus pneumoniae*.
